# Gear-up and recycle: an autophagy regulatory module boosts light usage in apple plants

**DOI:** 10.1093/plphys/kiag203

**Published:** 2026-04-11

**Authors:** Gunjan Sharma

**Affiliations:** Assistant Features Editor, Plant Physiology, American Society of Plant Biologists; School of Biosciences, University of Birmingham, Edgbaston B15 2TT, United Kingdom

“An apple a day keeps doctor away” is a familiar expression, highlighting the nutritional qualities of apples. Apple trees need light for photosynthesis to produce nutrient-rich fruits, but light availability becomes limited in crowded orchards, affecting fruit quality and production. Low-light perception and adaptation in plants require adjustments to leaf physiology and photosynthetic efficiency by alternating molecular signaling pathways.

Prolonged darkness causes energy depletion, which induces autophagy (Durgud et al. 2018). Autophagy literally means “eat your own” and is a highly conserved cellular pathway for degradation and recycling to maintain cellular homeostasis. During stress exposures, plants induce autophagy as an adaptive response (Gross et al. 2025). Low light induces autophagy to degrade impaired macromolecules and organelles to maintain cellular stability (Quilichini et al. 2019; Barros et al. 2017). However, our understanding of the molecular players connecting low-light responses and autophagy in apple growth and productivity is limited.

In a recently published article in *Plant Physiology*, Cheng et al. (2026) identified a link between low light availability and autophagy in apple plants. The authors initially demonstrated that low light induces autophagic activity based on the increased number of autophagosomes in apple leaves. In plants, a cysteine protease, ATG4, aids in autophagosome formation and the dynamic regulation of autophagy (Lu et al. 2019). There are 2 *ATG4* homologs (*MdATG4a* and *MdATG4b*) in apple; *MdATG4a* was induced by low-light stress. In low light, wild-type apple plants are smaller, with reduced height, leaf area, and petiole length, but when *MdATG4a* was overexpressed, the low-light grown plants were larger, suggesting that MdATG4a enhances responses to low light. Further investigations revealed that the low-light stress tolerance in *MdATG4a-*overexpressing plants is a result of increased photosynthetic efficiency, as demonstrated by the increased chlorophyll content, thicker leaf tissues, and photosynthetic parameter measurement. Additionally, *MdATG4a* overexpression increased autophagosome abundance, while knock-down plants had fewer autophagosomes.

Transcription factors regulate the autophagy pathway by modulating the expression of autophagy-associated target genes during stress responses and development in plants (Gross et al. 2025). Cheng et al. (2026) used a promoter fragment of *MdATG4a* as bait in a yeast one-hybrid assay, which identified a BASIC PENTACYSTEINE (BPC) transcription factor family member, MdBPC2, that suppresses induction of *MdATG4a* by direct binding to the C-box element in the promotor (Kooiker et al. 2005). *MdBPC2* overexpression in apple plants lowers MdATG4a expression and imparts sensitivity to low-light stress by reducing light use efficiency and suppressing autophagy. These observations confirm the suppressive role of MdBPC2 on MdATG4a-mediated autophagic responses and photosynthetic capacity.

The authors further found that MdBPC2 interacts with MdPIF3. Phytochrome-interacting factors (PIFs) are key regulators of response to light stimuli (Cai and Huq 2025). PIF3 is a negative regulator of light signaling and is stabilized in darkness (Liu et al. 2013). The interaction between MdBPC2 and MdPIF3 enhances the suppression of *MdATG4a* transcriptional induction.

In conclusion, Cheng and colleagues deciphered a mechanistic MdATG4a-MdBCP2-MdPIF3 module fine-tuning responses to light availability and autophagy. *MdATG4a* induction during low light heightens the autophagy pathway, conferring tolerance to low light through increased photosynthetic efficiency. However, *MdATG4a* is transcriptionally restricted by MdPIF3 and MdBPC2 during ample light to conserve cellular energy (Fig. 1). These results suggest that MdATG4a is a potential candidate for improving apple plant growth and fruit yield during low-light stress.

**Figure 1:**
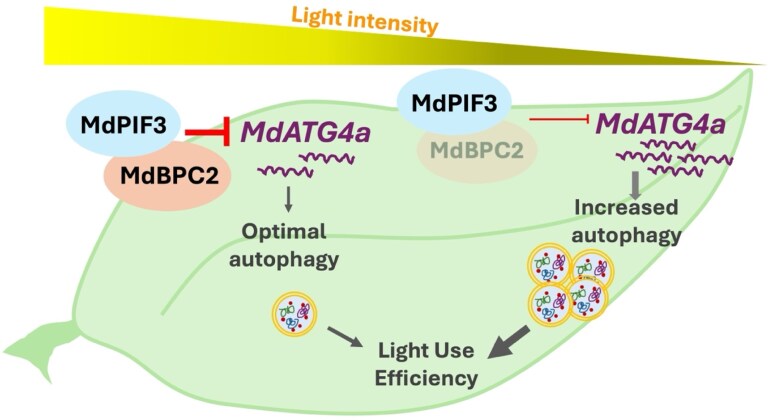
Proposed model for MdATG4a-MdBPC2-MdPIF3 module in regulating autophagy and light use efficiency during low-light stress. During optimal light intensity, expression of *MdATG4a* is predominantly suppressed by binding of MdBPC2 to the *MdATG4a* promotor itself and partly by interaction with MdPIF3. Reduced expression of *MdATG4a* leads to optimal autophagy and light use efficiency. During low-light stress, MdPIF3 is stabilized but the expression of *MdBPC2* is lowered, alleviating the repression of *MdATG4a* expression and autophagy. Increased autophagy improves light use efficiency, supporting nutrient-rich quality apple yield. Thick and thin arrows indicate increase and reduction, respectively (adapted and modified from Cheng et al. 2026).

## Related articles published in *Plant Physiology:*

He et al. (2025) revealed the HsfA1a-BAG5b module mediates thermotolerance by inducing *BAG5b* expression under high-temperature stress activating autophagy in tomato.

Ma et al. (2025) demonstrated that a central autophagy component ZmATG8c plays a role in conferring thermotolerance in maize.

## Data Availability

No new data was generated or analyzed for this article.
